# PDSS2‐Del2, a new variant of PDSS2, promotes tumor cell metastasis and angiogenesis in hepatocellular carcinoma via activating NF‐κB

**DOI:** 10.1002/1878-0261.12826

**Published:** 2020-11-04

**Authors:** Tingting Zeng, Zhi Tang, Lili Liang, Daqin Suo, Lei Li, Jiangchao Li, Yunfei Yuan, Xin‐Yuan Guan, Yan Li

**Affiliations:** ^1^ State Key Laboratory of Oncology in South China Collaborative Innovation Center for Cancer Medicine Sun Yat‐sen University Cancer Center Guangzhou China; ^2^ School of Public Health Guangdong Medical University Dongguan China; ^3^ Department of Clinical Oncology The University of Hong Kong China; ^4^ Vascular Biology Research Institute Guangdong Pharmaceutical University Guangzhou China

**Keywords:** angiogenesis, dimethyl fumarate, hepatocellular carcinoma, metastasis, nuclear factor‐κB, PDSS2‐Del2

## Abstract

Hepatocellular carcinoma (HCC) is among the leading causes of cancer‐related mortality worldwide. Our previous study identified a novel alternative splicing variant of prenyl diphosphate synthase subunit 2 (PDSS2) in HCC characterized by a deletion of exon 2, named PDSS2‐Del2, which is devoid of the tumor‐suppressive function of full‐length PDSS2 (PDSS2‐FL). To better understand the clinical significance of PDSS2‐Del2, we performed a BaseScope™ assay on an HCC tissue microarray and found that positive staining for PDSS2‐Del2 predicted a worse overall survival in patients with HCC (*P* = 0.02). PDSS2‐Del2 levels correlated significantly with microvessel counts in HCC tumor tissues. Importantly, PDSS2‐Del2 overexpression functionally promoted HCC metastasis, as demonstrated by *in vitro* and *in vivo* migration assays. *In vivo* assays also demonstrated that PDSS2‐Del2 increased angiogenesis in xenografts. Furthermore, we discovered that elevated PDSS2‐Del2 expression in HCC tumor cells decreased fumarate levels and activated the canonical nuclear factor‐κB pathway. The epithelial‐to‐mesenchymal transition (EMT) and WNT/β‐catenin signaling pathways were also activated by overexpression. Dimethyl fumarate (DMF), a fumaric acid ester, effectively reduced the metastasis induced by PDSS2‐Del2 as observed with *in vivo* spleen‐liver metastasis animal experiments. DMF is a prescribed oral therapy for multiple sclerosis and it might be a potential treatment for metastasis of patients with HCC. Early clinical trials are needed to validate its potential in this context.

## Graphical abstract

Unlike full‐length PDSS2, which has a tumor‐suppressive function, PDSS2‐Del2 promoted HCC metastasis and angiogenesis. Positive staining for PDSS2‐Del2 predicted a worse overall survival in patients with HCC. Elevated PDSS2‐Del2 expression in HCC tumor cells decreased fumarate levels and activated the canonical NF‐κB pathway. Dimethyl fumarate (DMF) might be a potential treatment for metastasis of patients with HCC.

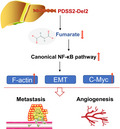

**Abbreviations**
ASH: alcoholic steatohepatitis
CoQ10: coenzyme Q10
CoQ10H2: coenzyme Q10 (reduced form)
DMF: dimethyl fumarate
EMT: epithelial‐to‐mesenchymal transition
FH: fumarate hydratase
HCC: hepatocellular carcinoma
HE: hematoxylin and eosin
IHC: immunohistochemistry
NASH: nonalcoholic steatohepatitis
NF‐κB: nuclear factor κB
PDSS2: prenyl diphosphate synthase subunit 2
PDSS2‐Del2: exon 2‐deleted PDSS2
PDSS2‐FL: full‐length PDSS2
qRT‐PCR: quantitative reverse transcription PCR
shRNA: short hairpin RNA
TMA: tissue microarray

## 1 Introduction

Liver cancer is the fifth most common human malignancy worldwide, with an increasing incidence every year, and is the second leading cause of cancer‐related death globally [1]. Hepatocellular carcinoma (HCC) represents the major histologic type of primary liver cancer, accounting for 70–85% of all liver cancer cases [2]. The major cause of HCC in Southeast Asia is chronic hepatitis B virus infection. In sub‐Saharan Africa, the main risk factor is exposure to aflatoxin‐contaminated food. In Japan and developed countries in North America and Europe, epidemiologic evidence connecting the disease is hepatitis C virus infection, alcoholic liver disease, type 2 diabetes, obesity, metabolic disorders and non‐alcoholic fatty liver diseases [3, 4, 5, 6]. Despite major advances in the diagnostic management of HCC, only one‐third of newly diagnosed patients are at present eligible for curative treatments [7]. The 5‐year survival rate after tumor resection for early stage HCC varies from 17 to 53% [8, 9]. However, there is still a high incidence of postoperative recurrence and a high likelihood of intrahepatic metastasis [10, 11]. Therefore, it is crucial to obtain in‐depth knowledge of the mechanisms driving HCC metastasis, as this knowledge could contribute substantially to HCC treatment.

In our previous study, we showed that downregulation of prenyl diphosphate synthase subunit 2 (PDSS2), a key enzyme in coenzyme Q10 (CoQ10) synthesis, plays a key role in hepatocellular carcinogenesis [12]. The PDSS2 deficiency was found to induce a metabolic shift from mitochondrial respiration to aerobic glycolysis and chromosomal instability and ultimately to induce malignant transformation of the immortalized human liver cell line MIHA [12]. We also found a novel splicing variant of PDSS2: PDSS2‐Del2 (in which alternative splicing deleted exon 2) in HCC cells. The polyprenyl synthesis domain of PDSS2 is disrupted in PDSS2‐Del2, and CoQ10 synthesis does not proceed. An *in vivo* xenograft formation assay suggested that tumor suppressive ability was absent in PDSS2‐Del2 cells but not in PDSS2‐FL (full‐length PDSS2) cells. Here, we found that PDSS2‐Del2 could increase HCC cell motility and tumor metastasis. Our results also revealed that PDSS2‐Del2 increased angiogenesis *in vivo*. Identifying PDSS2‐Del2 and exploring the signaling events that promote metastasis and angiogenesis may provide new insights for therapeutic targeting of HCC. Our research also found that dimethyl fumarate (DMF), a prescribed oral therapy for relapsing‐remitting multiple sclerosis, might be a potential treatment for metastasis of HCC patients with PDSS2‐Del2 increases.

## 2 Materials and methods

### 2.1 Cell lines, plasmids and short hairpin RNA (shRNA)

The HCC cell lines SMMC7721, BEL7402 and PLC8024 were obtained from the Institute of Virology, Chinese Academy of Medical Sciences (Beijing, China). Huh7 was purchased from American Type Culture Collection (Manassas, VA, USA). The cells were confirmed by cytogenetics as being of human origin. Cells were cultured in DMEM with 10% FBS and incubated at 37 °C in a humidified incubator containing 5% CO_2_. The lentiviral construct plenti6‐PDSS2‐Del2 and a lentiviral packaging mix were purchased from GeneCopoeia (Guangzhou, China). Short hairpin RNA targeting PDSS2‐Del2 was purchased from Sigma‐Aldrich (St. Louis, MO, USA). Short hairpin RNA targeting p65‐nuclear factor‐κB (NF‐κB) were purchased from GenePharma (Suzhou, China).

### 2.2 HCC clinical specimens, tissue microarray and BaseScope™ detection assay

Primary HCC tumors and corresponding nontumor tissues were collected at Sun Yat‐sen University Cancer Center. The studies were conducted in accordance with recognized ethical guidelines of the Declaration of Helsinki. The study using human tissues was reviewed and approved by the Committees for Ethical Review of Research involving Human Subjects of Sun Yat‐sen University Cancer Center. Written informed consent was obtained from the patients. RNA was extracted from 128 pairs of HCC tumor and nontumor tissues and used for quantitative PCR (qPCR) assay. The ages of the patients ranged from 20 to 76 years at the time of surgery (median age: 52 years) and the male : female ratio was 8 : 1.

A tissue microarray (TMA) block (containing 143 pairs of HCC tumor and nontumor tissues) was constructed as previously described [13]. The ages of patients ranged from 27 to 83 years at the time of surgery (median age: 52 years). The BaseScope^TM^ probe specific for PDSS2‐Del2 was designed by ACD (Advanced Cell Diagnostics, Newark, CA, USA). *In situ* hybridization to visualize single molecules of PDSS2‐Del2 in the HCC TMA was performed with the BaseScope^TM^ detection reagent kit in accordance with the guidelines provided by the manufacturer (ACD)[14]. The BaseScope^TM^ probe specific for PDSS2‐Del2 was validated by testing on positive and negative controls (Fig. S2). The results were scanned using an Automated Quantitative Pathology Imaging System (Vectra, Perkin Elmer, Waltham, MA, USA). We subjected the signal data to ROC curve analysis (SPSS) with respect to overall survival and PDSS2‐Del2 staining was considered positive when the signals ≥ 30 per tissue dot.

### 2.3 Quantitative PCR

Total RNA was extracted from tissues using TRIzol (Invitrogen, Carlsbad, CA, USA) and reverse transcription was performed using SuperScript III (Invitrogen). The primers were designed as shown in Fig. S3. Quantitative PCR was performed using SYBR Green Supermix and a Light Cycler 480 (Roche, Basel, Switzerland). PCR products were subjected to dissociation curve analysis to exclude amplification of nonspecific products. Quantitative PCR data were processed using the δCt method.

### 2.4 Cell motility assay

Cells suspended in serum‐free DMEM were seeded into chambers with an 8‐µm microporous filter (BD, Bedford, MA, USA). The cells were attracted with DMEM with 10% FBS. Twenty‐four hours later, the cells were fixed, stained with crystal violet and counted. Three independent replicate assays were conducted.

### 2.5 Animal experiments

All animal procedures were approved by the Institutional Animal Care and Use Committee of Sun Yat‐sen University Cancer Center. Four‐ to six‐week‐old BALB/c nude mice were used in the study. SMMC7721 derivative cells (PDSS2‐Del2‐overexpressing cells and vector control cells, 4 × 10^6^) were inoculated subcutaneously into both flanks of athymic mice (*n* = 6). The xenograft growth was monitored for 1 month. Then the animals were sacrificed and xenografts were isolated and fixed in 10% formalin.

BEL7402 and SMMC7721 derivative cells (PDSS2‐Del2‐overexpressing cells and vector control cells) were injected into the tail vein (BEL7402: 1 × 10^6^, SMMC7721: 8 × 10^5^) or footpad (BEL7402: 5 × 10^5^, SMMC7721: 3 × 10^5^) of athymic mice (*n* = 6 for each group). The animals were sacrificed 60 days later and the lungs, livers and hearts examined. For the footpad‐injected groups, the popliteal lymph nodes were also isolated and fixed in 10% formalin.

Intrasplenic model was also established to assess the *in vivo* metastasis ability. PLC8024 derivative cells (Del2 and vector control, 1 × 10^6^) or Huh7 derivative cells (2 × 10^6^) were transplanted through intrasplenic injection into the 4‐ to 6‐week‐old male nude mice (*n* = 9 for PLC8024 and *n* = 8 for Huh7). At the end of the experiments, the animals were euthanized and tumor metastatic nodules formed on the liver surfaces were counted. Livers and lungs were fixed and HE (hematoxylin and eosin) staining was performed.

### 2.6 Fumarate supplementation assay

PLC8024 derivative cells (PDSS2‐Del2 or vector control, 2 × 10^6^) were injected intrasplenically into nude mice as described above. Dimethyl fumarate and methyl cellulose were purchased from Sigma‐Aldrich. At 12 days post surgery, mice from each group were randomized into either vehicle control or DMF groups. Mice were gavaged daily with vehicle (0.8% methyl cellulose) or DMF (30 mg/kg suspended in 0.8% methyl cellulose) [15]. At the end of the experiments, the animals were euthanized and experiments were performed as described above.

### 2.7 Staining for F‐actin and immunofluorescence

Cells were fixed in 4% formaldehyde, stained with fluorescent phalloidin conjugate (Sigma‐Aldrich) and counterstained with 4’,6’‐diamidino‐2‐phenylindole (DAPI; Invitrogen, Carlsbad, CA, USA). Images were acquired with an Olympus BX51.

Cells plated on coverslips were fixed with 4% paraformaldehyde and permeabilized with 0.1% Triton X‐100 at 4 °C for 10 min. After blocking, the cells were stained with β‐catenin antibody (Cell Signaling Technology, Danvers, MA, USA) and counterstained with DAPI (Invitrogen, OR). Images were captured with a confocal microscope Olympus FV1000 or Olympus BX51.

### 2.8 CoQ10 detection

CoQ10 and its redox state were assessed using ultrahigh‐performance liquid chromatography tandem mass spectrometry as described in a previous study [16]. A total of 5 × 10^6^ cells were collected, frozen in liquid nitrogen and then subjected to mass spectrometry analysis. Eight independent samples were assessed for each group.

### 2.9 Liquid chromatography coupled with Orbitrap mass spectrometry

A total of 5 × 10^6^ cells were cultured without serum for 24 h and then cultured in DMEM with 10% FBS for 12 h. Then, the cells were frozen in liquid nitrogen and subjected to analysis. The metabolites were separated and analyzed using an Ultimate 3000 UHPLC (Dionex, Sunnyvale, CA, USA) coupled with a Q Exactive^TM^ Hybrid Quadrupole‐Orbitrap Mass Spectrometer (QE‐MS, Thermo Fisher Scientific, Wilmington, DE, USA) system. An Acquity HSS T3 column (100 × 2.1 mm i.d., 1.8‐µm particle size, Waters) was used in the study. The Orbitrap Q Exactive‐MS was equipped with an HESI probe.

### 2.10 Western blotting and immunohistochemistry

Western blotting was performed according to a standard protocol and the antibodies are listed in the supplementary data. Chemiluminescence signals were captured by film exposure or Quantity One system (Bio‐Rad, Hercules, CA, USA). Immunohistochemistry (IHC) was performed using the standard streptavidin‐biotin‐peroxidase complex method with the following antibodies: CKpan antibody, anti‐human CD34 and VEGF, and anti‐mouse CD34. Detailed information is listed in the supplementary data.

### 2.11 Microvessel counts

The tissue microarray section was stained with anti‐human CD34 antibodies (GeneTech, China). The tissue microarray was scanned with Vectra 2 TMA Scanning System (Perkin‐Elmer). The microvessels formed in each tissue dot were counted under a 20× objective. Microvessel counts ≥ 90 were designated high microvessel counts and counts < 90 low microvessel counts (Fig. S4). The results were assessed by two independent pathologists. If the results were consistent, the results were chosen. Where results differed, the two pathologists discussed and agreed on an appropriate outcome.

### 2.12 Statistical analyses

The data are expressed as mean ± standard error of the mean (SEM). Statistical analyses were carried out using the spss software package (SPSS 16.0, SPSS Inc., Chicago, IL, USA). Quantification of IF and IHC was performed using image j software (NIH Image, Bethesda, MD, USA) (https://imagej.nih.gov/ij/). Correlations between PDSS2‐Del2 and the clinical features were assessed with a Pearson’s Chi‐square test. A survival curve was generated by Kaplan–Meier analysis. A log‐rank test was used to compare different survival curves. Multivariate survival analyses were performed using the Cox regression model. Differences between two groups were examined with Student’s *t*‐test. *P* < 0.05 was considered statistically significant.

## 3 Results

### 3.1 PDSS2‐Del2 positive staining predicts worse survival of HCC patients

**Fig. 1:**
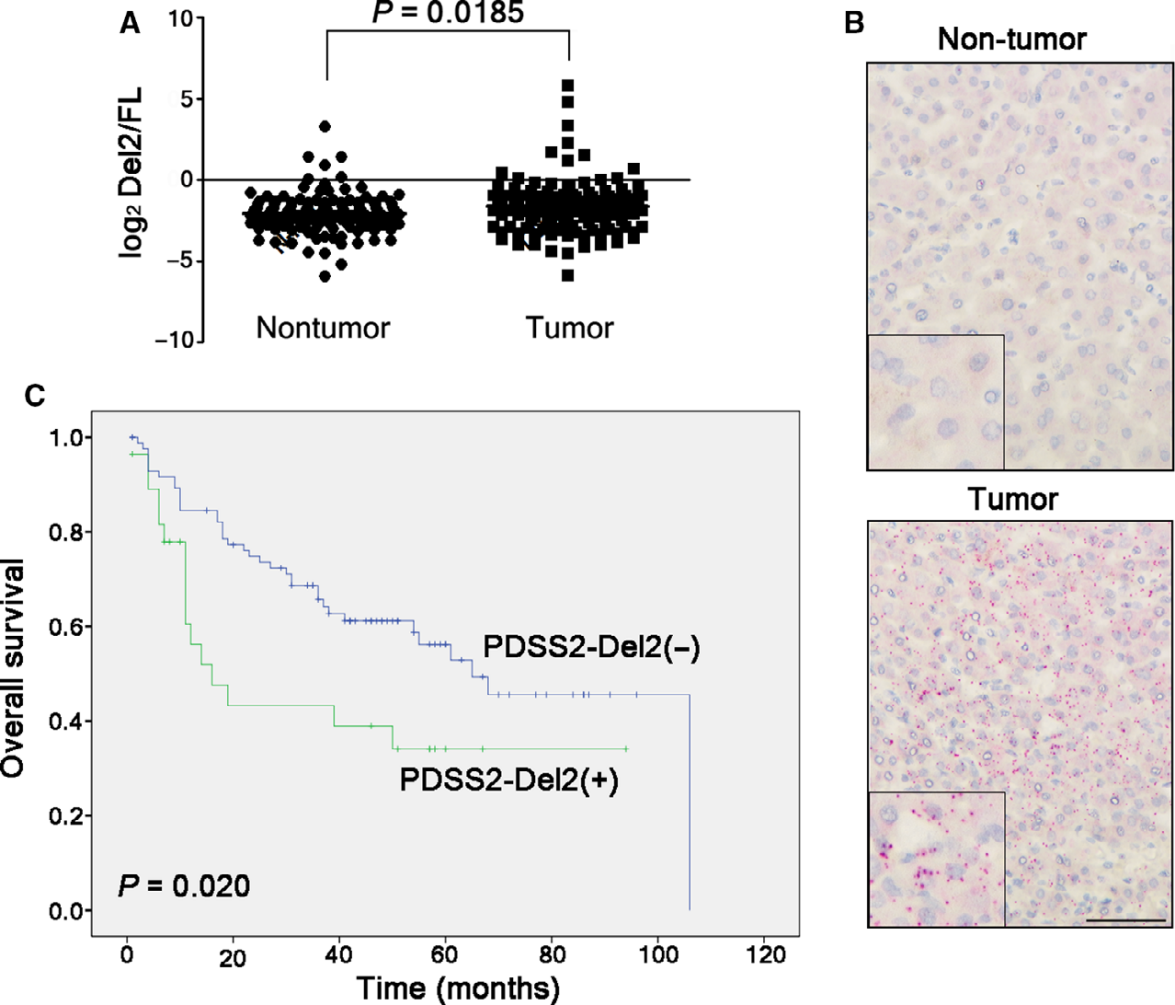
PDSS2‐Del2 expression increases in HCC tumor tissues. (A) The PDSS2‐Del2/FL ratio (with exon 2 deletion/without exon 2 deletion) was compared between HCC tumor tissues and paired nontumor tissues. (B) Representative pictures of PDSS2‐Del2 detected by the BaseScope™ probe in HCC tumor tissue compared with nontumor tissue (bottom right corner: magnified picture) (scale bar: 50 µm). (C) The survival curves showed that PDSS2‐Del2 positive staining predicted a worse overall survival for HCC patients (Kaplan–Meier method).

We detected PDSS2‐Del2 and PDSS2‐FL in 128 pairs of HCC tumor tissues and their corresponding nontumor tissues with qPCR. The ratio of PDSS2‐Del2/FL was significantly increased in HCC tumor tissues compared with nontumor tissues (*P* = 0.0185, Fig. 1A). In 51/128 cases (39.84%), the Del2/FL ratio in tumor tissues was more than twice the ratio in paired nontumor tissues (T_Del2/FL_/N_Del/FL_> 2). *In situ* hybridization to visualize individual PDSS2‐Del2 molecules was performed on an HCC tissue microarray using a BaseScope™ assay. Positive staining for PDSS2‐Del2 was detected in 5.31% (6/113) of nontumor tissues, whereas the positive staining increased to 24.56% (28/114) in HCC tumor tissues (*P* < 0.01, Fig. 1B). The correlation study showed that PDSS2‐Del2 positive staining correlated with tumor stage (*P* = 0.008) and tumor embolus formation (*P* = 0.001) (Table 1). PDSS2‐Del2 positive staining predicted a worse overall survival in HCC patients, as determined by Kaplan–Meier analysis (*P* = 0.020, Fig. 1C). However, Cox regression analysis demonstrated that PDSS2‐Del2 positive staining was not an independent prognostic factor for the overall survival of patients with HCC (Table S1).

**Table 1:** Correlation analysis of PDSS2‐Del2 with clinicopathological features of patients with HCC

Clinicopathologic features	PDSS2‐Del2		*P*‐value^a^
	Negative	Positive	
*Gender*			
Female	4 (100%)	0	0.245
Male	82 (74.55%)	28 (25.45%)	
*Age*			
≤ 57	59 (73.75%)	21(26.25%)	0.521
> 57	27 (79.41%)	7 (20.59%)	
*HBsAg*			
−	15 (93.75%)	1 (6.25%)	0.066
+	71 (72.45%)	27 (27.55%)	
*Stage*			
I–II	58 (84.06%)	11 (15.94%)	**0.008**
III–IV	28 (62.22%)	17 (37.78%)	
*Cirrhosis*			
0–1	41 (82.00%)	9 (18.00%)	0.150
2–3	45 (70.31%)	19 (29.69%)	
*Tissue invasion*			
−	68 (77.27%)	20 (22.73%)	0.403
+	18 (69.23%)	8 (30.77%)	
*Tumor embolus*			
−	76 (81.72%)	17 (18.28%)	**0.001**
+	10 (47.62%)	11 (52.38%)	
*Steatohepatitis*			
No	74 (76.29%)	23 (23.71%)	0.680
ASH	1 (50.00%)	1 (50.00%)	
NASH	11 (73.33%)	4 (26.67%)	

ASH, alcoholic steatohepatitis; NASH, nonalcoholic steatohepatitis.

PDSS2‐Del2 staining was considered positive when signal points were ≥ 30 in the BaseScope™ detection assay.

^a^ Pearson’s Chi‐square; cirrhosis: 0, no; 1, mild; 2, moderate; 3, severe.

### 3.2 PDSS2‐Del2 promotes cell motility *in vitro*

**Fig. 2:**
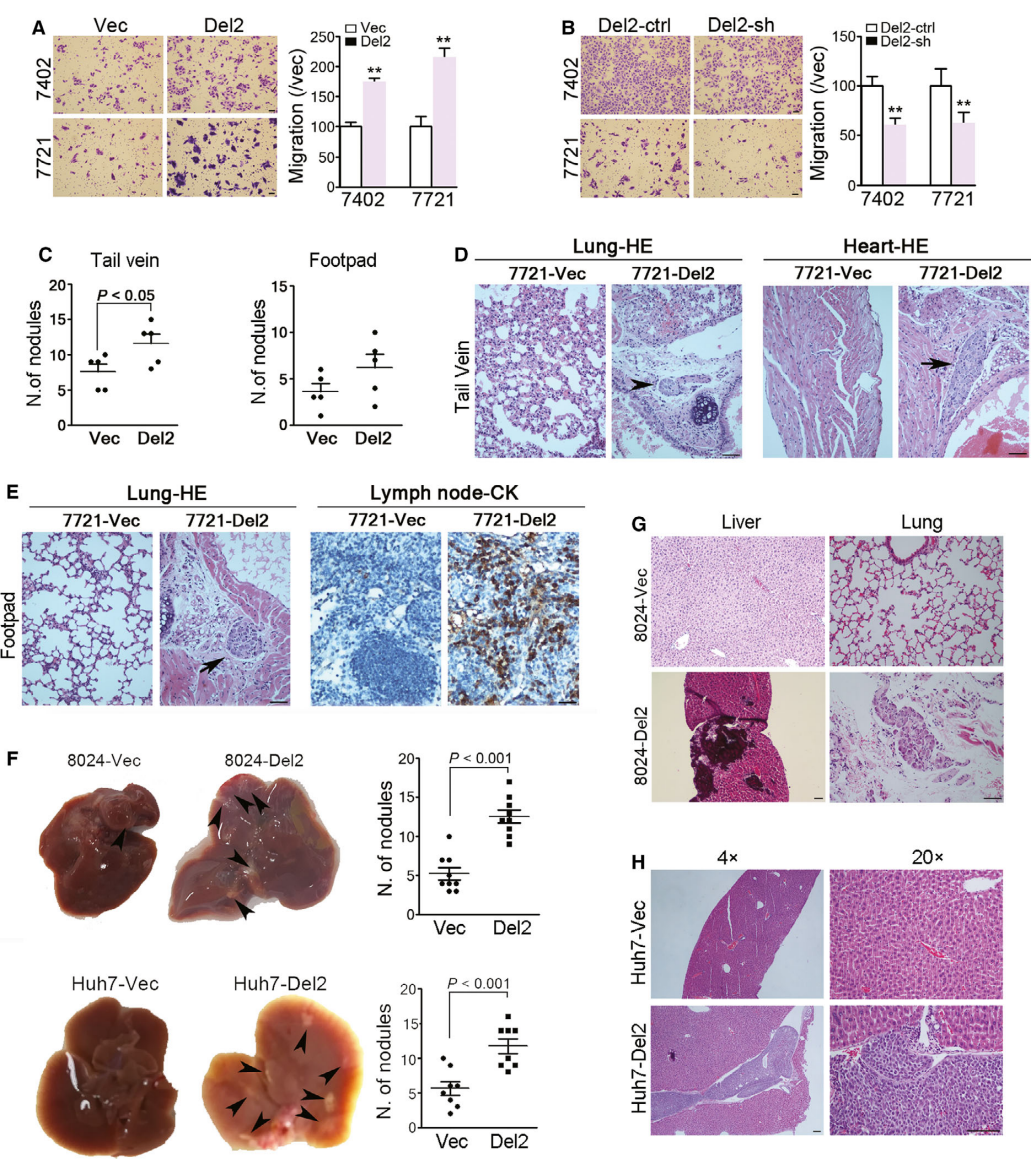
PDSS2‐Del2 promotes HCC cell metastasis. (A) Representative pictures and summary of migrated BEL7402 and SMMC7721 derivative cells (PDSS2‐Del2 and vector control) (scale bar: 50 µm). The results are shown as the mean ± SEM of three independent experiments. (B) Representative pictures and summary of migrated PDSS2‐Del2 cells transduced with shRNA and scramble control (ctrl). The results are shown as the mean ± SEM of three independent experiments (scale bar: 50 µm). (C) The number of metastatic nodules on the surface of the livers increased in the 7721‐PDSS2‐Del2 group compared with the vector control (7721‐Vec) in both the hematogenous (tail vein) and lymph node (footpad) metastasis models. (D) HE staining of lungs and hearts of the 7721‐PDSS2‐Del2 and vector control groups in the hematogenous metastasis models (scale bar: 50 µm) (top). (E) HE staining of lungs and CK immunostaining of lymph nodes in the lymph node metastasis models (scale bar: 50 µm) (bottom). (F–H) The *in vivo* experimental metastatic assay was performed by intrasplenic injection of 8024‐Del2, Huh7‐Del2 and the corresponding control cells to nude mice. (F) Representative images of liver and the numbers of nodules formed on the surface of the livers were summarized (arrowheads: tumor metastatic nodules). (G) HE staining of metastatic tumor cells in liver or lung of 8024‐derivative cells (scale bar: 50 µm). (H) HE staining of metastatic tumor cells in liver of Huh7‐derivative cells (scale bar: 100 µm).

To test the effect of PDSS2‐Del2 on HCC tumor cell motility, an *in vitro* cell migration assay was performed. The results demonstrated that PDSS2‐Del2 overexpression significantly increased cell motility. The number of PDSS2‐Del2‐overexpressing cells that migrated through the filter increased significantly compared with the number of vector control cells (Fig. 2A). When overexpressed PDSS2‐Del2 was knocked down, the number of migrated cells decreased compared with that of the scramble control cells (ctrl) (Fig. 2B).

### 3.3 PDSS2‐Del2 promotes cell metastasis *in vivo*

We next examined whether PDSS2‐Del2 could promote cell metastasis *in vivo*. SMMC7721 derivative cells (7721‐Del2 and ‐Vec) were inoculated into the tail vein or subcutaneously into the footpad of nude mice (*n* = 5 for each group). Two months later, animals were sacrificed and surface nodules on the liver and lungs were compared between the group injected with PDSS2‐Del2‐overexpressing cells and the group injected with control cells. In both the hematogenous (tail vein) and lymph node (footpad) metastasis models, the number of metastatic nodules on the liver increased in the PDSS2‐Del2 group (Fig. 2C). HE staining also showed that the number of tumor colonies in the lungs of the PDSS2‐Del2 group increased in both the hematogenous and lymph node metastasis models (Fig. 2D,E). In the hematogenous metastasis model, we also observed surface nodules in the hearts of the PDSS2‐Del2 group (2/5 mice), whereas no metastatic nodules formed in the control group (0/5 mice) (Fig. 2D). In the lymph node metastasis model, the lymph nodes were isolated, fixed and immunostained with CK. The results demonstrated that the number of positively stained cells increased in the PDSS2‐Del2 group compared with the vector control group (Fig. 2E). BEL7402 derivative cells were also injected into the tail vein (*n* = 6) of nude mice. Similar results were obtained in the lymphatic metastasis model (Fig. S1).

The spleen‐liver metastasis experiments were performed to validate further the metastasis potential of PDSS2‐Del2. The number of metastatic tumor nodules on liver increased significantly in the PLC8024‐Del2 group compared with the vector control group (*P* < 0.001, Fig. 2F,G). In addition, metastatic tumor cells were observed in the lung section of 5/9 mice of PLC8024‐Del2 compared with only 1/9 in the control group (Fig. 2G). The result was further confirmed by repeating the experiments with Huh7 derivative cells (Fig. 2F,H).

### 3.4 PDSS2‐Del2 activates NF‐κB

**Fig. 3:**
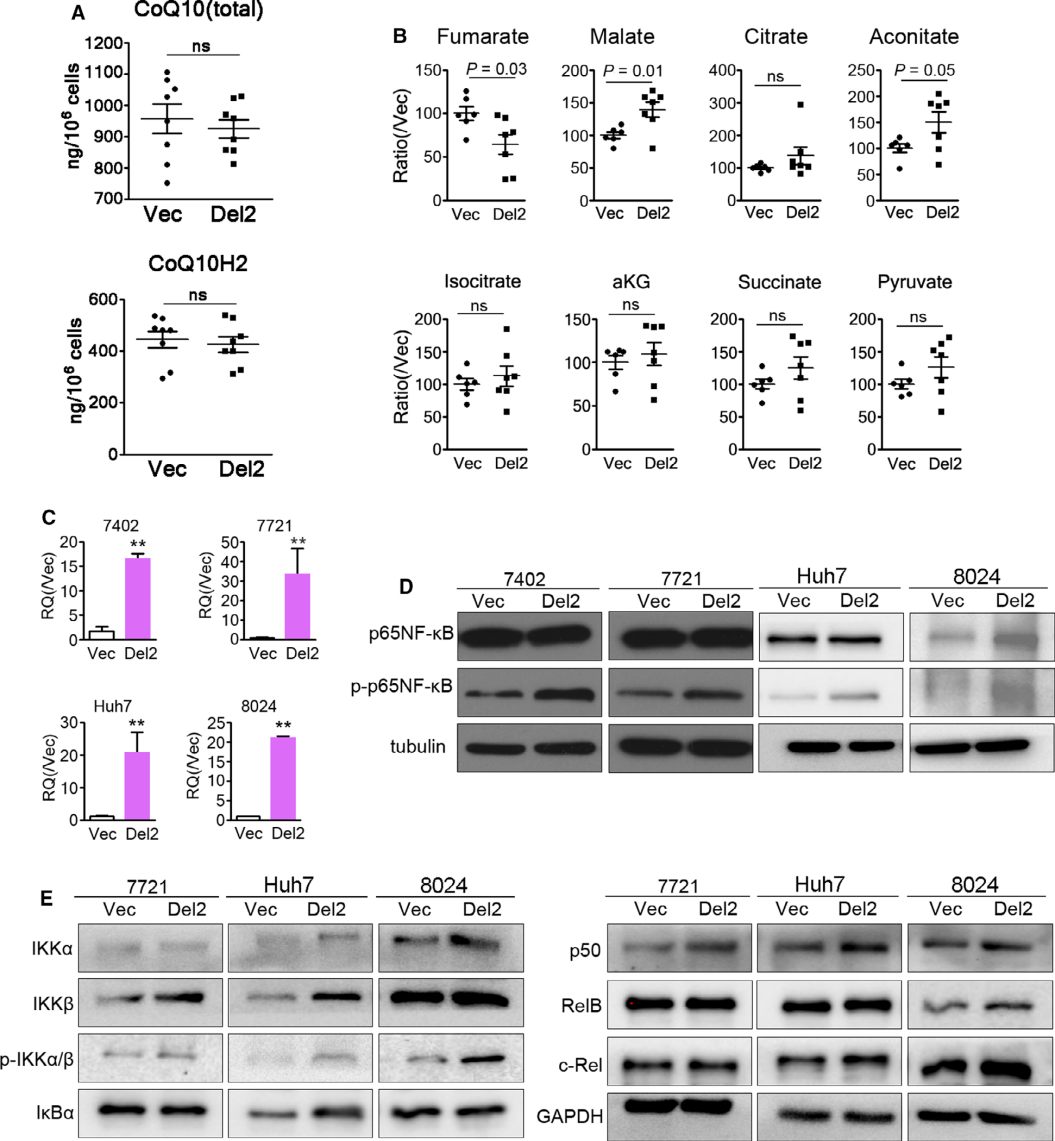
PDSS2‐Del2 induces NF‐κB activation. (A) CoQ10 (total) and CoQ10H2 (reduced form of CoQ10) levels were determined in 7721‐PDSS2‐Del2 cells and vector control cells by ultra‐high‐performance liquid chromatography tandem mass spectrometric analysis (ns: not significant). (B) Fold changes of TCA cycle intermediates (fumarate, malate, citrate, aconitate, isocitrate, aKG and succinate) and pyruvate in 7721‐PDSS2‐Del2 cells and 7721‐Vec cells (aKG, α‐ketoglutarate; ns, not significant). (C) The relative quantification of PDSS2‐Del2 was evaluated by qRT‐PCR in Del2‐overexpressing cells. (D) NF‐κB p65 and phospho‐NF‐κB p65 (ser536) were detected by western blotting in PDSS2‐Del2‐overexpressing cells. Tubulin was set as a loading control. (E) IKKα, IKKβ, phosphor‐IKKα/β (ser176/180), IκBα, NF‐κB p50, RelB and c‐Rel were detected by western blotting. GAPDH was set as a loading control.

According to the sequence analysis, the polyprenyl synthesis domain of PDSS2 is disrupted in PDSS2‐Del2. Ultrahigh‐performance liquid chromatography tandem mass spectrometry was performed on PDSS2‐Del2 derivative cells. PDSS2‐Del2 introduction did not affect CoQ10 level or status (reduced form CoQ10H2) (Fig. 3A). We found that PDSS2‐Del2 overexpression decreased fumarate (*P* < 0.05) and increased malate levels (*P* < 0.05) and aconitate marginally (*P* = 0.05), whereas citrate, isocitrate, α‐ketoglutarate, succinate and pyruvate levels remained unchanged (Fig. 3B). Because dimethyl fumarate was reported to be associated with blockade of NF‐κB activation [15, 17, 18], we tested NF‐κB level and found that the phosphorylated NF‐κB p65 (ser536) increased in Del2‐overexpressing cells (Fig. 3C,D). We further tested the NF‐κB signaling pathway and found that Del2 overexpression in HCC cells induced increases of IKKα, IKKβ, phosphorylation of IKKα/β (ser176/180) and NF‐κB p50 (Fig. 3E). Slight increase of c‐Rel was also observed in Del2‐overexpressing Huh7 and PLC8024 cells (Fig. 3E). Taken together, these results indicate that canonical NF‐κB pathway is activated in PDSS2‐Del2‐overexpressing HCC cells.

### 3.5 PDSS2‐Del2 activates the WNT/β‐catenin pathway

**Fig. 4:**
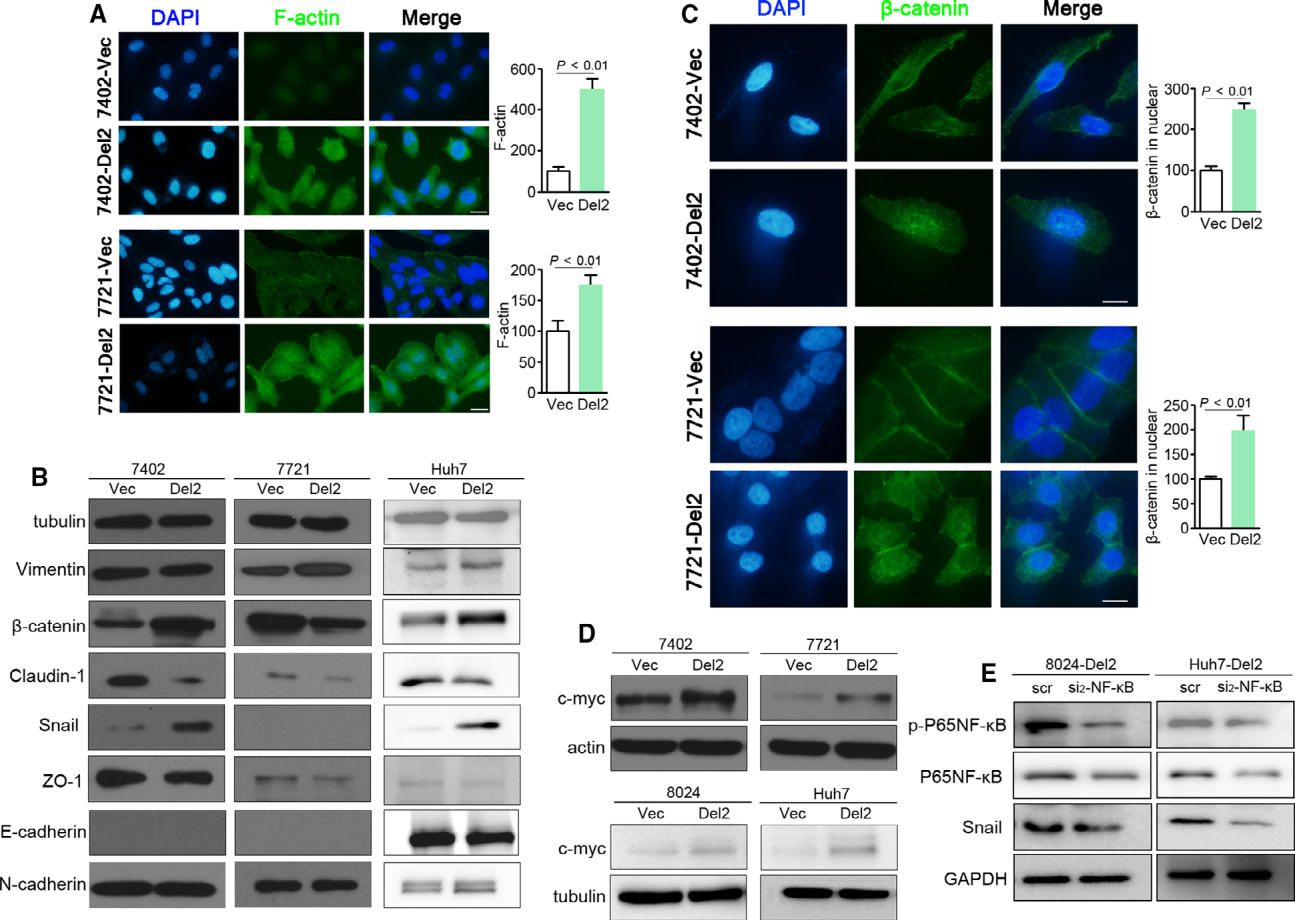
PDSS2‐Del2 promotes EMT and activates the WNT/β‐catenin pathway. (A) Representative pictures and summary of F‐actin staining of PDSS2‐Del2‐overexpressing BEL7402 and SMMC7721 cells compared with vector control cells (Vec) (blue: DAPI; green: F‐actin) (scale bar: 50 µm) (*t*‐test). The fluorescence of F‐actin was quantified using image j software and relative quantification was calculated (*right*). (B) The epithelial‐ and mesenchymal‐related proteins were detected by western blotting in PDSS2‐Del2‐overexpressing cells compared with vector control cells. Tubulin was set as a loading control. (C) Representative pictures and summary of IF demonstrated that β‐catenin translocated into the nuclei in PDSS2‐Del2‐overexpressing cells (blue: DAPI; green: β‐catenin) (scale bar: 10 µm) (*t*‐test). The fluorescence of β‐catenin in nuclei was quantified using image j software and relative quantification was calculated (*right*). (D) The protein level of c‐myc was determined by western blotting. Actin or tubulin was set as loading control. (E) The protein levels of phosphorylated NF‐κB P65, NF‐κB P65 and Snail were detected in PDSS2‐Del2‐overexpressing cells treated with siRNA targeting NF‐κB P65.

Because PDSS2‐Del2 promotes HCC cell motility, we next examined the cells with F‐actin staining. Stress fiber staining was increased in PDSS2‐Del2‐overexpressing BEL7402 and SMMC7721 cells compared with vector control cells (Fig. 4A). Furthermore, we examined the protein levels of epithelial‐ and mesenchymal‐related proteins in PDSS2‐Del2‐overexpressing cells and vector control cells. In the transduced HCC cell lines, Claudin‐1 was downregulated in PDSS2‐Del2‐overexpressing cells. Snail increased in two PDSS2‐Del2‐overexpressing HCC cell lines. Slight decrease of E‐cadherin and increase of N‐cadherin were observed in Huh7‐Del2 cells (Fig. 4B). β‐catenin increased in BEL7402 and Huh7 whereas it decreased in SMMC7721 with PDSS2‐Del2 overexpression (Fig. 4B). However, immunofluorescence (IF) results revealed that nuclear translocation of β‐catenin increased in SMMC7721 as in BEL7402 cells with PDSS2‐Del2 overexpression (Fig. 4C). We next examined the c‐myc which is a target of β‐catenin activation. As shown in Fig. 4D, PDSS2‐Del2 overexpression increased c‐myc significantly in PDSS2‐Del2 derivative cells. Furthermore, knocking‐down p65 NF‐κB resulted in significant decrease of Snail protein level in PDSS2‐Del2‐overexpressing cells (Fig. 4E), confirming the importance of NF‐κB in the pathway activation.

### 3.6 PDSS2‐Del2 increases angiogenesis

**Fig. 5:**
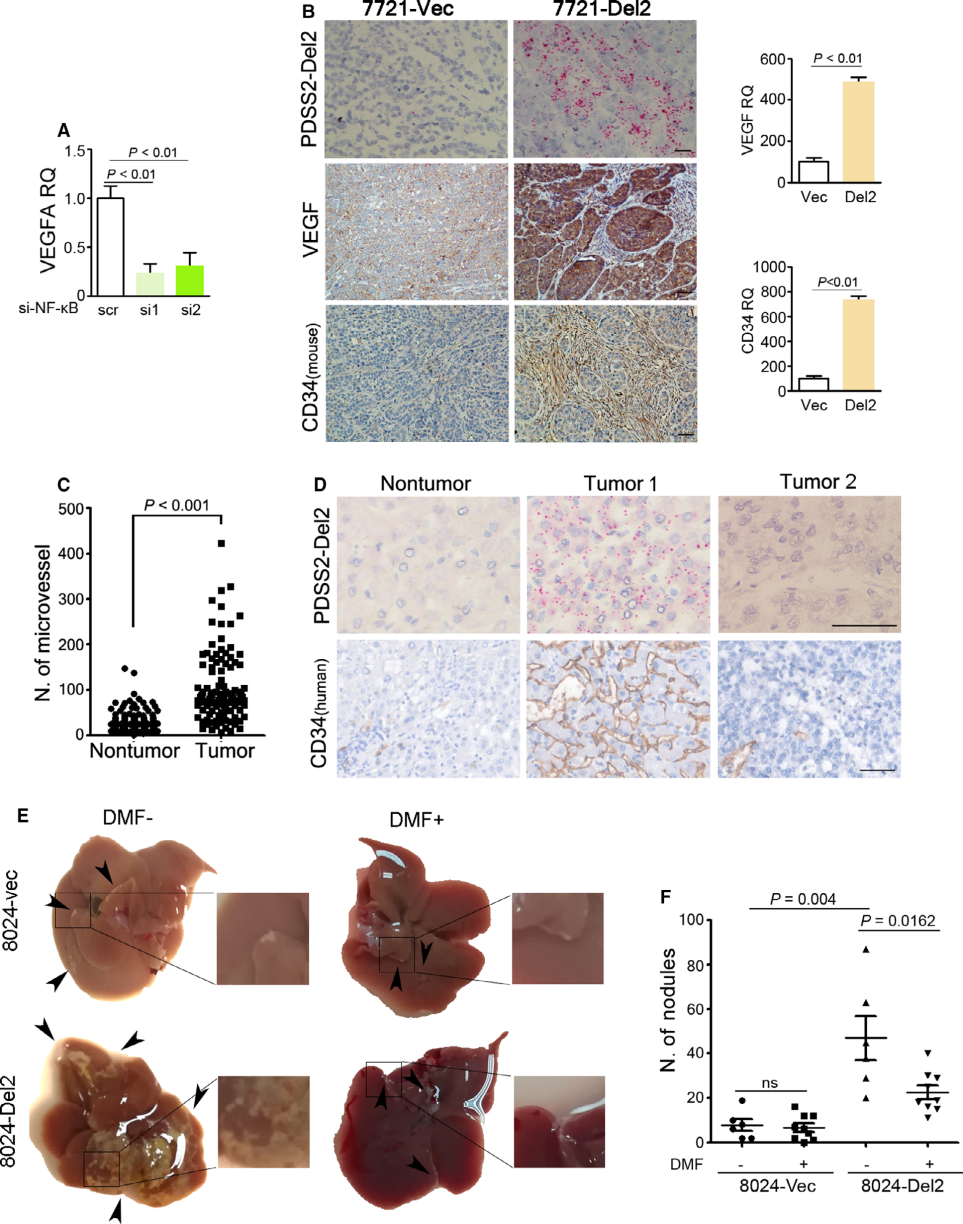
PDSS2‐Del2 increases angiogenesis in HCC and fumarate supplementation decreases metastasis induced by PDSS2‐Del2. (A) 8024‐Del2 cells were treated with siRNAs targeting p65 NF‐κB and VEGFA was detected using qPCR. (B) Representative pictures of PDSS2‐Del2 detected by the PDSS2‐Del2 BaseScope™ probe (upper), VEGF and CD34 detected by IHC (middle and bottom) in xenografts induced by PDSS2‐Del2‐overexpressing 7721 cells (7721‐Del2) and vector control cells (7721‐Vec) (scale bar: 50 µm). The staining results of VEGF and CD34 (including intensity and area) were quantified using image j software and relative quantification was calculated (right). (C) CD34 staining of the HCC TMA indicated that the number of microvessels was increased in tumor tissues compared with nontumor tissues. (D) Representative pictures of PDSS2‐Del2 detected with the BaseScope™ probe and CD34 staining in HCC tumor tissues and nontumor tissues (scale bar: 50 µm). (E,F) 2 × 10^6^ 8024‐Del2 and vector control cells were injected intrasplenically and mice then treated with 0.8% methyl cellulose (vehicle) or DMF, respectively. The mice were separated into four groups: 8024‐Vec‐Vehicle (DMF^–^), 8024‐Vec‐DMF (DMF^+^), 8024‐Del2‐Vehicle (DMF^–^) and 8024‐Del2‐DMF (DMF^+^). (E) Representative pictures of liver isolated from each group (black arrows indicate the nodules formed on the surface of the livers). The boxed regions are amplified as images shown in righthand panels. (F) The numbers of metastatic nodules formed on the surface of the livers are summarized.

NF‐κB is one of main transcription factors related to the induction of angiogenesis [19]. Knocking‐down p65 NF‐κB also decreased the RNA level of VEGFA in 8024‐Del2 cells (Fig. 5A). We next examined vessel formation in xenografts formed in nude mice. Vascular endothelial growth factor (VEGF) levels increased significantly in PDSS2‐Del2‐overexpressing xenografts (Fig. 5B). A significant increase in CD34 immunohistochemical staining was observed in PDSS2‐Del2‐overexpressing xenografts (Fig. 5B).

We next examined microvessel formation in an HCC tissue microarray using CD34 immunostaining. The microvessel counts were significantly increased in HCC tumor tissues (104.4 ± 7.057, *n* = 113) compared with nontumor tissues (31.86 ± 2.199, *n* = 118) (*P* < 0.001) (Fig. 5C). In the 92 tumor cases with both PDSS2‐Del2 and microvessel count information, the PDSS2‐Del2 positive expression in patients with higher microvessel counts (≥ 90) was significantly higher than that in patients with lower microvessel counts (< 90) (36.84% vs. 12.96%, *P* < 0.01) (Fig. 5D, Table 2). The correlation analysis indicated that PDSS2‐Del2 expression correlated significantly with microvessel counts in HCC tumor tissues (*R* = 0.280, *P* = 0.007, Table 2).

**Table 2:** Correlation analysis of PDSS2‐Del2 and microvessels in HCC tumor tissues

		Microvessel < 90	Microvessel ≥ 90	total
PDSS2‐Del2	Negative	47	24	71
	Positive	7	14	21
Total		54	38	92
		*R* = 0.280, *P* = 0.007^a^		

^a^ Pearson correlation analysis.

### 3.7 Fumarate supplementation inhibits metastasis induced by PDSS2‐Del2

Because fumarate decreased in PDSS2‐Del2 cells (Fig. 3B), we were wondering whether fumarate supplementation could inhibit tumor cell metastasis. Dimethyl fumarate (DMF) is an anti‐inflammatory drug already in clinical use for multiple sclerosis and psoriasis [15, 17]. So DMF was administered orally to mice daily after 12 days post intrasplenic injection models were established. In the 8024‐Del2 group that was treated with DMF, the number of metastatic nodules on the surface of liver decreased significantly compared with the group treated with vehicle only, whereas no significant decrease was observed in vector control cells between DMF supplementary group and vehicle group (Fig. 5E,F).

## 4 Discussion

Of all primary liver cancers, hepatocellular carcinoma is the most common neoplasm, accounting for most cases [2, 20]. Around 85% of HCC are developed as a result of chronic hepatitis caused by HBV, HCV or non‐alcoholic steatohepatitis [21]. Although advances in prognosis have been made in recent years, for patients in advanced stages of the disease, treatment options are still limited. Due to the early metastasis, fast tumor growth and multidrug resistance, the 5‐year survival rate of HCC is very low [22]. A more comprehensive understanding of the HCC metastasis will facilitate the development of therapeutic strategies for reducing the HCC cancer‐related death rate.

PDSS2 is an important enzyme in CoQ10 synthesis. Studies including our previous report demonstrated that PDSS2 is an important tumor suppressor in the development of malignant melanoma and gastric cancer [23, 24]. In addition, we found that different variants of PDSS2 exist in HCC tumor tissues and cell lines [12]. Of note, one of the variants, PDSS2‐Del2, exhibits a different function than that of PDSS2. We used a novel ultrasensitive *in situ* hybridization approach (BaseScope™) [14] to detect PDSS2‐Del2 RNA molecules in an HCC TMA. Survival analysis suggested that PDSS2‐Del2 is a predictor of a worse overall survival. PDSS2‐Del2 positive staining was correlated with tumor stage and tumor embolus formation. Microvascular tumor embolism is an independent predictor of HCC recurrence after liver transplantation [25]. The presence of portal vein and/or microvascular tumor embolus increases significantly in HCC patients with intrahepatic metastasis compared with that in patients with multicentric occurrence [26]. Furthermore, mesenchymal circulating tumor cells are significantly correlated with the presence of embolus or microembolus in HCC patients [27]. This literature led us to wonder whether PDSS2‐Del2 could promote HCC tumor cell metastasis.

Because the functional domain of the enzyme is disrupted in PDSS2‐Del2, it loses the ability to synthesize CoQ10. Neither the quantity nor the status (as a reduced or oxidized form) of CoQ10 changed in PDSS2‐Del2‐overexpressing cells. The tumor‐suppressive function of PDSS2 was absent in PDSS2‐Del2‐overexpressing cells. Interestingly, both *in vitro* and *in vivo* assays verified that HCC cells overexpressing PDSS2‐Del2 could acquire enhanced migration motility. In the *in vitro* migration assay, we used shRNA targeting PDSS2‐Del2 in the Del2‐overexpressing HCC cells. The shRNA also targets PDSS2‐FL (full length). Considering the low ratio of PDSS2‐FL and loss of function of PDSS2‐FL in parental HCC cells [12], we believe that the knockdown of PDSS2‐Del2 accounts for most of the migration inhibition effect in PDSS2‐Del2 overexpressing HCC cells.

Our results demonstrated that the TCA cycle metabolite fumarate decreased significantly in PDSS2‐Del2 cells. Dimethyl fumarate has been reported in many studies to inhibit the NF‐κB pathway [15, 17]. We therefore tested the protein levels of the NF‐κB pathway and found that canonical NF‐κB signaling was activated in PDSS2‐Del2‐overexpressing cells. EMT is a crucial process in embryonic development and is utilized by tumor cells to gain mobility and invasiveness during tumor metastasis [28]. It is characterized by loss of cell–cell junctions, cytoskeletal remodeling, morphological changes and acquisition of migratory and invasive capabilities [29]. The EMT process involves a shift in regulation of a cohort of genes, for example, transcription repressors such as snail are upregulated [30] and some genes are downregulated [31]. In the present study, PDSS2‐Del2 introduction increases the transcription factor snail and decreases Claudin, which is the most diverse component of tight junctions [31] and plays critical roles in maintaining cell–cell integrity and in regulating paracellular ion transport [32]. Further analysis revealed that β‐catenin accumulated in the nuclei and that the WNT pathway was activated, leading to increased c‐myc in PDSS2‐Del2‐overexpressing cells.

NF‐κB is a major transcription factor related to the induction of inflammation, angiogenesis and cancer‐related processes such as cell proliferation, apoptosis and metastasis [19, 33]. NF‐κB can stimulate transcription of proliferation regulating genes, genes involved in metastasis, VEGF‐dependent angiogenesis and cell immortality by telomerase [34]. NF‐κB inhibitor IMD0354 can reduce expression of VEGFA and disrupt corneal angiogenesis [35]. Parthenolide, another NF‐κB inhibitor, leads to downregulation of hypoxia‐dependent angiogenesis by preventing NF‐κB activation in colorectal cancer cells [36]. HCC is a highly vascularized malignancy with strong angiogenetic activity and the VEGF pathway has been targeted as a treatment for HCC [37, 38]. Here, NF‐κB seemed to present a basal molecular mechanism influencing angiogenesis in PDSS2‐Del2‐overexpressing xenografts. In addition, the HCC clinical sample analysis confirmed a positive correlation between neovascularization and PDSS2‐Del2 expression.

Though the loss of fumarate hydratase (FH) and subsequent accumulation of fumarate marks the genetic cancer syndrome Hereditary leiomyomatosis and renal cell cancer (HLRCC) [39, 40, 41], it was reported that dimethyl fumarate, a fumaric acid ester, could effectively abrogate NF‐κB‐dependent mammosphere formation of breast cancer cells and inhibit xenograft tumor growth [15]. DMF can also inhibit tumor growth and metastasis in cutaneous T‐cell lymphoma [17]. DMF is an anti‐inflammatory drug which is applied for the treatment of relapsing‐remitting multiple sclerosis [42]. It is neuro‐protective, executing anti‐inflammation via inhibition of NF‐κB and activation of Nrf2 pathways [43, 44]. DMF can also modulate immune reactions without significant immune suppression [45]. Our research suggests that fumarate decreased significantly in PDSS2‐Del2 cells and DMF supplementation could effectively attenuate the metastasis ability of HCC cells endowed by PDSS2‐Del2, whereas no significant change was observed in vector control cells. Taken together, our results suggest that DMF may have therapeutic potential for inhibiting metastasis of HCC patients with PDSS2‐Del2 overexpression.

## 5 Conclusions

During cancer progression, metastasis and angiogenesis have been shown to significantly affect patient prognoses. Here, we show that PDSS2‐Del2, a new transcript variant of PDSS2, plays various roles in HCC metastasis and angiogenesis. These observations suggest that PDSS2‐Del2 may be an important oncogene in HCC. Overall, our study reveals the multifaceted role of PDSS2‐Del2, which is different from that of full‐length PDSS2, in HCC metastasis and angiogenesis. We also identify PDSS2‐Del2 as a new target for HCC treatment, and DMF supplementation might be a potential therapeutic treatment for metastasis of HCC patients with PDSS2‐Del2 increases.

## Conflict of interest

The authors declare no conflict of interest.

## Author contributions

TZ, ZT, LL, DS and LL performed the experiments. JL and YY provided technical support and important samples. YL initiated the study, analyzed data and wrote the manuscript. XG revised the manuscript. All authors read and accepted the manuscript.

## Supporting information


**Table S1.** Univariate and multivariate analysis of different prognostic variables in patients with HCC.

**Fig. S1.** PDSS2‐Del2 increases HCC cell metastasis *in vivo*.

**Fig. S2.** BaseScope™ assay is validated.

**Fig. S3.** The schematic diagram of primers designed for exon2 deletion or non‐deletion of PDSS2 detection.

**Fig. S4.** Representative pictures of CD34 staining.
