# Supplementary material for: PDSS2‐Del2, a new variant of PDSS2, promotes tumor cell metastasis and angiogenesis in hepatocellular carcinoma via activating NF‐κB
Source: Mol Oncol. 2020 Nov 4;14(12):3184–97. doi: 10.1002/1878-0261.12826 (PMC7718950; doi:10.1002/1878-0261.12826)
Supplement: Supplementary file 1 — Table S1. Univariate and multivariate analysis of different prognostic variables in patients with HCC. [file MOL2-14-3184-s001.docx]

**Supporting information**:

**PDSS2-Del2, a new variant of PDSS2, promotes tumor cell metastasis and angiogenesis in hepatocellular carcinoma via activating NF-κB**

**Primers and sequences:**

| Del2 detection primers: | |
| --- | --- |
| PDSS2-(587-721 lo)-F1 | 5’ ACCACAGCCAGTCAAAG 3’ |
| PDSS2-R10 | 5’ CTTGGTGTTCTGTAGCAGAG 3’ |
| PDSS2 (FL) detection primers: | |
| PDSS2-lo-F1 | 5’ CTTGTACATGACAGCTGG 3’ |
| PDSS2-lo-R1 | 5’ ATGAGTAGATCCCACTGACC 3’ |
| 18S-F-r | 5’ CTCTTAGCTGAGTGTCCCGC 3’ |
| 18S-R-r | 5’ CTGATCGTCTTCGAACCTCC 3’ |
| actin-F | 5’ GGCATTCACGAGACCACCTAC 3’ |
| actin-R | 5’ CGACATGACGTTGTTGGCATAC 3’ |
| shRNA target Del2 sequence | GATCACGGAGCTAATTCAT |

**Antibodies:**

| **Antibody** | **Company** | **Catalog #** |
| --- | --- | --- |
| β-tubulin | Cell Signaling Technology | #2128 |
| actin | Cell Signaling Technology | #4970 |
| vimentin | Cell Signaling Technology | #3390 |
| β-catenin | Cell Signaling Technology | #8480 |
| ZO-1 | Cell Signaling Technology | #5406 |
| E-cadherin | Cell Signaling Technology | #14472 |
| claudin-1 | Cell Signaling Technology | #13255 |
| snail | Cell Signaling Technology | #3879 |
| N-cadherin | Cell Signaling Technology | #4061 |
| NF-κB p65 | Cell Signaling Technology | #8242 |
| phospho- NF-κB p65 (ser536) | Cell Signaling Technology | #3033 |
| IKKα | Cell Signaling Technology | #11930 |
| IKKβ | Cell Signaling Technology | #8943 |
| Phospho-IKKα/β (ser176/180) | Cell Signaling Technology | #2697 |
| IκBα | Cell Signaling Technology | #4814 |
| RelB | Cell Signaling Technology | #4922 |
| c-Rel | Cell Signaling Technology | #12707 |
| NF-κB1 p105/p50 | Cell Signaling Technology | #12540 |
| GAPDH | Santa Cruz biotechnology | SC-59541 |
| c-myc | Cell Signaling Technology | #5605 |
| CKpan | Abcam | ab6401 |
| human CD34 | GeneTech | GM716529 |
| VEGF | GeneTech | GT217002 |
| mouse CD34 | Santa Cruz Biotechnology | sc-52478 |

**Supplementary table:**

**Supplementary table 1: Univariate and multivariate analysis of different prognostic variables in patients with HCC**

| **Clincopathological features** | **Univariate analysis** | | **Multivariate analysis** | |
| --- | --- | --- | --- | --- |
|  | **HR (95% CI)** | ***P*-value** | **HR (95% CI)** | ***P*-value** |
| Gender | 2.127 (0.520-8.702) | 0.294 |  |  |
| Age | 0.540 (0.298-0.976) | 0.041 | 0.674 (0.356-1.276) | 0.226 |
| HBsAg | 3.824 (1.200-12.191) | 0.023 | 3.751 (0.873-16.119) | 0.076 |
| Stage | 1.576 (0.966-2.572) | 0.068 | 1.520 (0.838-2.755) | 0.168 |
| cirrhosis | 1.632 (0.985-2.702) | 0.057 | 1.694 (0.921-3.117) | 0.090 |
| Tissues invading | 1.732 (1.004-2.986) | 0.048 | 1.467 (0.767-2.806) | 0.247 |
| Tumor embolus | 4.614 (2.637-8.074) | <0.001 | 3.815 (1.894-7.683) | <0.001 |
| PDSS2-Del2 | 1.990 (1.098-3.605) | 0.023 | 1.178 (0.620-2.238) | 0.618 |

HR, hazard ratio; CI, confidence interval.

**Supplementary figure legends:**

**Supplementary figure 1: PDSS2-Del2 increases HCC cell metastasis *in vivo*.**

**A**) Representative pictures of HE staining and summary of tumor cell colonies formed in lungs in the hematogenous model induced by BEL7402 derivative cells (7402-Del2 and 7402-Vec). The boxed regions are amplified as the pictures on the right (original magnification: 100×). **B**) The number of colonies formed in the lungs is summarized.

**Supplementary figure 2: BaseScope assay is validated.**

The xenografts were induced by injecting PDSS2-Del2 overexpressing cells (7721-Del2) and vector control cells (7721-Vec) into the flanks of nude mice, respectively. Then the xenografts were isolated, fixed and paraffin-embedded. BaseScope probe was validated by performing the assay on negative control (7721-Vec) and positive control (7721-Del2) (original magnification: 200×).

**Supplementary figure 3: The schematic diagram of primers designed for exon2 deletion or no-deletion of PDSS2 detection.**

**A**) Primers detecting exon2 non-deletion locate in the exon2 region (FL, full length). **B**) The forward primer detecting exon2 deletion locate at the junction of exon1 and exon3. (E, exon; F, forward primer; R, reverse primer)

**Supplementary figure 4: Representative pictures of CD34 staining.**

The microvesel counts were recorded by CD34 positive staining of HCC TMA. Representative pictures of HCC tumor tissues of low microvessel counts (low) and high microvessel counts (high) are displayed (original magnification: 200×).
