# Supplementary figures and images for: PDSS2‐Del2, a new variant of PDSS2, promotes tumor cell metastasis and angiogenesis in hepatocellular carcinoma via activating NF‐κB
Source: Mol Oncol. 2020 Nov 4;14(12):3184–97. doi: 10.1002/1878-0261.12826 (PMC7718950; doi:10.1002/1878-0261.12826)

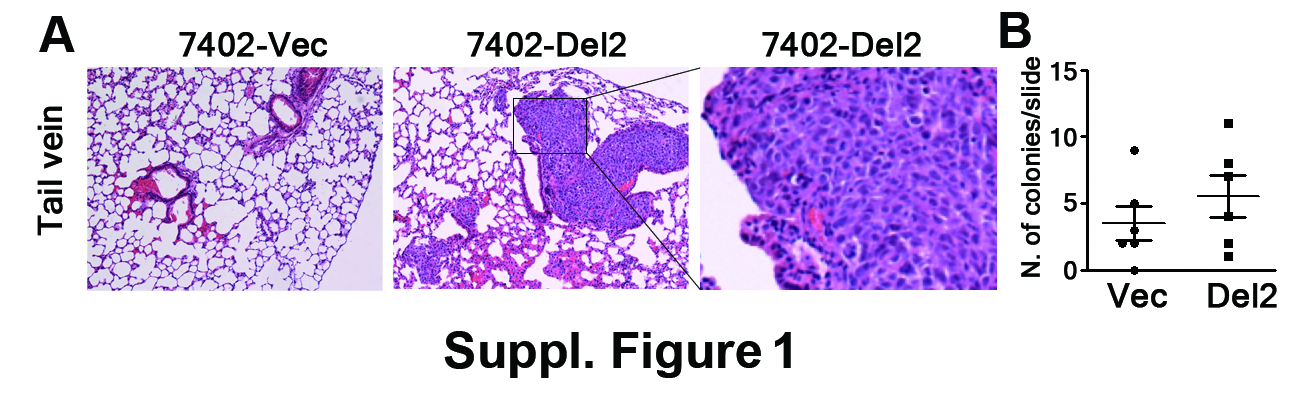

Supplement: Supplementary file 2 — Fig. S1. PDSS2‐Del2 increases HCC cell metastasis in vivo. [file MOL2-14-3184-s002.tif]

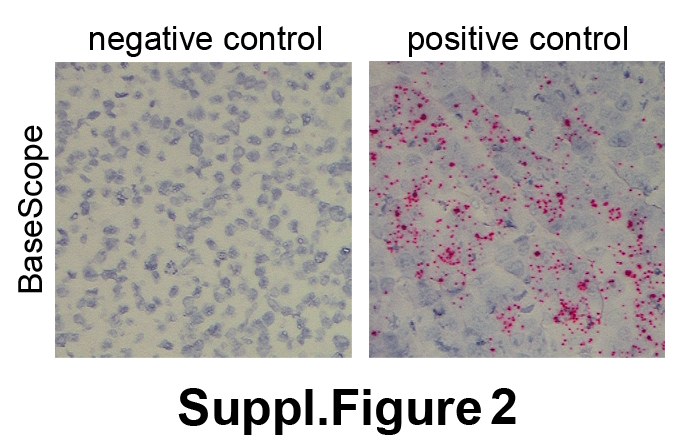

Supplement: Supplementary file 3 — Fig. S2. BaseScope™ assay is validated. [file MOL2-14-3184-s003.tif]

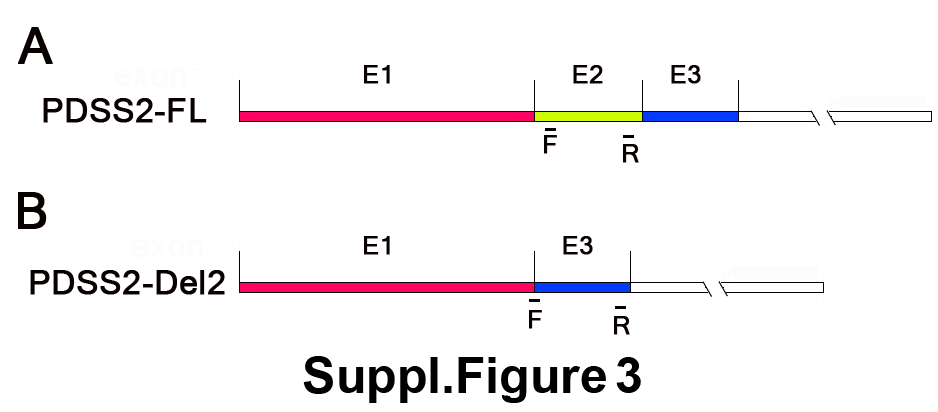

Supplement: Supplementary file 4 — Fig. S3. The schematic diagram of primers designed for exon2 deletion or non‐deletion of PDSS2 detection. [file MOL2-14-3184-s004.tif]

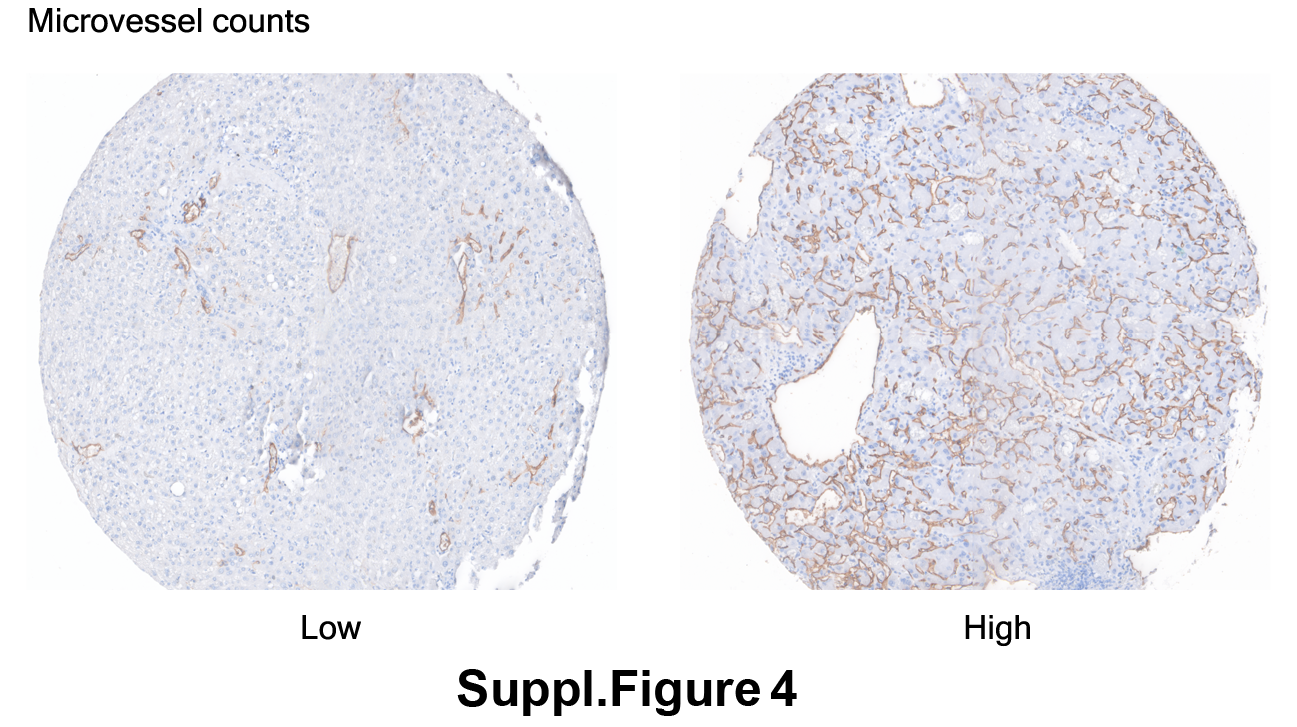

Supplement: Supplementary file 5 — Fig. S4. Representative pictures of CD34 staining. [file MOL2-14-3184-s005.tif]
